# Targeting Ezh2 could overcome docetaxel resistance in prostate cancer cells

**DOI:** 10.1186/s12885-018-5228-2

**Published:** 2019-01-08

**Authors:** Xiaofu Qiu, Wei Wang, Bijun Li, Bo Cheng, Kangjian Lin, Jian Bai, Huanhui Li, Guosheng Yang

**Affiliations:** 1 Department of Urology, Southern Medical University Third Medical College, Guangzhou, 510317 China; 2 Department of Urology, Guangzhou General Hospital of Guangzhou Military Command, Guangzhou, 510515 China; 3 Department of Urology, the Second Affiliated Hospital of Guangdong Medical College, Zhanjiang, 524003 China; 4 Department of Urology, the Affiliated Hospital of Southwest Medical University, Luzhou, 646000 China; 5 Department of Urology, Guangdong Second Provincial General Hospital, Guangzhou, 510317 China

## Abstract

**Background:**

Docetaxel was used to treat metastatic CRPC patients. However, Doc resistance in prostate cancer (PCa) hinders its clinical application.

**Objective:**

To understand the underlying mechanisms by which Doc resistance is developed and to find novel therapeutic target to cure Doc resistant PCa has clinical importance.

**Methods:**

We established Doc resistant cell lines and explored the role of Ezh2 in the development of Doc resistance by overexpressing its cDNA or using its inhibitor.

**Results:**

We found that Ezh2 was induced in our established Doc resistant (DocR) cells, which was attributable to the silenced expression of miR-101-3p and miR-138-5p. Blockage of Ezh2 activity by either inhibitor or miRNA mimics could overcome Doc resistance by suppressing Doc-induced cancer stem cells populations. Mechanistically, Ezh2 activity was required for the induced expression of Nanog, Sox2 and CD44 upon Doc treatment.

**Conclusions:**

Targeting Ezh2 could overcome Doc resistance.

**Electronic supplementary material:**

The online version of this article (10.1186/s12885-018-5228-2) contains supplementary material, which is available to authorized users.

## Introduction

Prostate cancer (PCa) remains the 2nd most lethal disease for males worldwide [1]. Androgen receptor (AR) is implicated into the onset and progression of PCa [2–4], thus the primary treatment for PCa is androgen deprivation therapy (ADT) by either surgical castration or chemical castration. Although ADT can efficiently suppress tumor growth, the castration resistant prostate cancer (CRPC) will invariably occur after approximate 2-year treatment [5]. In addition to AR, various molecules can also provide growth advantages to CPRC.

Docetaxel (Doc), which was approved by FDA in 2014 to treat metastatic CRPC, significantly improves patients’ survival [6, 7]. As a chemotherapy drug, Doc can stabilize microtubule structure by binding β-tubulin, leading to the impaired cell division [8]. For this reason, cells undergo mitotic arrest and apoptosis [9, 10]. However, Doc resistance has already become one of the clinical problems. Clinically, about 50% of patients poorly responded to Doc treatment and patients who initially responded it well would eventually develop resistance phenotype [6, 11, 12]. Therefore, understanding the development of Doc resistance and finding novel therapy to overcome it become major scientific and clinical interests.

As one important component of polycomb-repressive complex 2 (PRC2), Ezh2 suppresses gene expression via catalyzing histone 3 lysine 27 tri-methylation [13]. Ezh2 has been 5documented as an oncogene in various cancers [14]. Elevated EZH2 expression is correlated with development of castration-resistant prostate cancer (CRPC) [15, 16], but the mechanisms by which EZH2 drives up PCa development is still elusive. Even though the importance of epigenetic regulation by Ezh2 via PRC2 complex exists in primary and metastatic PCa, the non-epigenetic regulation of Ezh2 was also involved in the progression of CRPC [15]. For instance, Ezh2 could interact and methylate AR to regulate specific gene expression, offering survival signals to CRPC cells [15]. Furthermore, the role of Ezh2 in the homeostatic regulation of cancer stem cells has also been recognized [17, 18]. All these suggest Ezh2 may become potential target for CPRC patients.

In this study, we first identified that Ezh2 was overexpressed in LNCaP and CWR22Rv1 Doc resistant cells (LNCaP DocR and CWR22Rv1 DocR) compared to their parental cells, which was attributable to the downregulation of miR-101-3p and miR-185-5p. Importantly, inhibition of Ezh2 by its specific inhibitor (DZNEP) or these two miRNAs mimics could re-sensitize DocR cells to Doc treatment while overexpression of Ezh2 was sufficient to confer Doc resistance to PCa cells. Our data reinforce the importance of Ezh2 in the development of Doc resistance and suggest targeting Ezh2 may improve the efficacy of Doc treatment.

## Materials and methods

### Cell culture

LNCaP and CWR22Rv1 cells were purchased from Cell Bank of Chinese Academy Of Science (Shanghai, China) and were maintained in RPMI-1640 Medium supplemented with 10% FBS (Gibco), penicillin (100 units/ml), streptomycin (100 μg/ml) and 1% L-glutamine. All cell lines were cultured in a 5% CO2 humidified incubator at 37 °C. MiRNA mimic (10 nM,Qiagen) with their corresponding negative controls were introduced into cells using the Lipofectamine 2000 (Invitrogen) following the manufacturer’s instructions.

### In vitro DocR cell line establishment

LNCaP cells and CWR22Rv1 were continuously adding various concentration of Doc for more than 6 months. Then cells were maintained with fixed Doc after 6 month: LNCaP DocR cells were maintained in 5 nM Doc while CWR22Rv1 DocR cells were kept in 20 nM Doc.

### Western blotting

Cells were lysed in RIPA buffer. 20 μg protein was loaded and separated on 10% SDS/PAGE gel. Samples were transferred onto PVDF membranes (Millipore). After being blocked in 5% milk for 1 h, the membranes were probed with specific primary antibodies overnight at 4 °C: Ezh2 (D2C9, Cell signaling), GAPDH (SC-32233, Santa Cruz). After 3 times extensive wash, blots were incubated with HRP-conjugated secondary antibody for 1 h at room temperature before the chemiluminescent reaction.

### RNA isolation and real time PCR

Trizol reagent (Invitrogen) was used to isolate total RNA. cDNA was made using Superscript III reverse transcription system (Invitrogen) from 1 μg RNA. Quantitative real-time PCR (qRT-PCR) was performed using a Bio-Rad CFX96 system with SYBR green to determine the interested mRNA expression. Primers are as follows:

Nanog (forward), 5′- TTTGTGGGCCTGAAGAAAACT-3′;

Nanog (reverse), 5’-AGGGCTGTCCTGAATAAGCAG-3′;

Sox2 (forward), 5’-TGGACAGTTACGCGCACAT -3′;

Sox2 (reverse), 5’-CGAGTAGGACATGCTGTAGGT-3′;

CD44 (forward), 5’-CTGCCGCTTTGCAGGTGTA-3′;

CD44 (reverse), 5’-CATTGTGGGCAAGGTGCTATT-3′;

Ezh2 (forward), 5’-AATCAGAGTACATGCGACTGAGA-3′;

Ezh2 (reverse), 5’-GCTGTATCCTTCGCTGTTTCC-3′;

GAPDH (forward), 5’-AATGGACAACTGGTCGTGGAC-3′;

GAPDH (reverse), 5’-CCCTCCAGGGGATCTGTTTG-3′.

### Sphere formation assay

5 × 10^3^ cells were suspended in serum free RPMI and equally mixed with growth factor enriched matrigel. 100 μL mixture was seeded into 24-well plate and supplemented with 1 mL medium. After two weeks later, floating cells were counted under microscopic machine.

### Statistics

Differences in mean values between two groups were analyzed by two-tailed Student’s *t* test. *p* ≤ 0.05 was considered as statistically significant.

## Results

### Ezh2 was required and sufficient to cause doc resistance

**Fig. 1:**
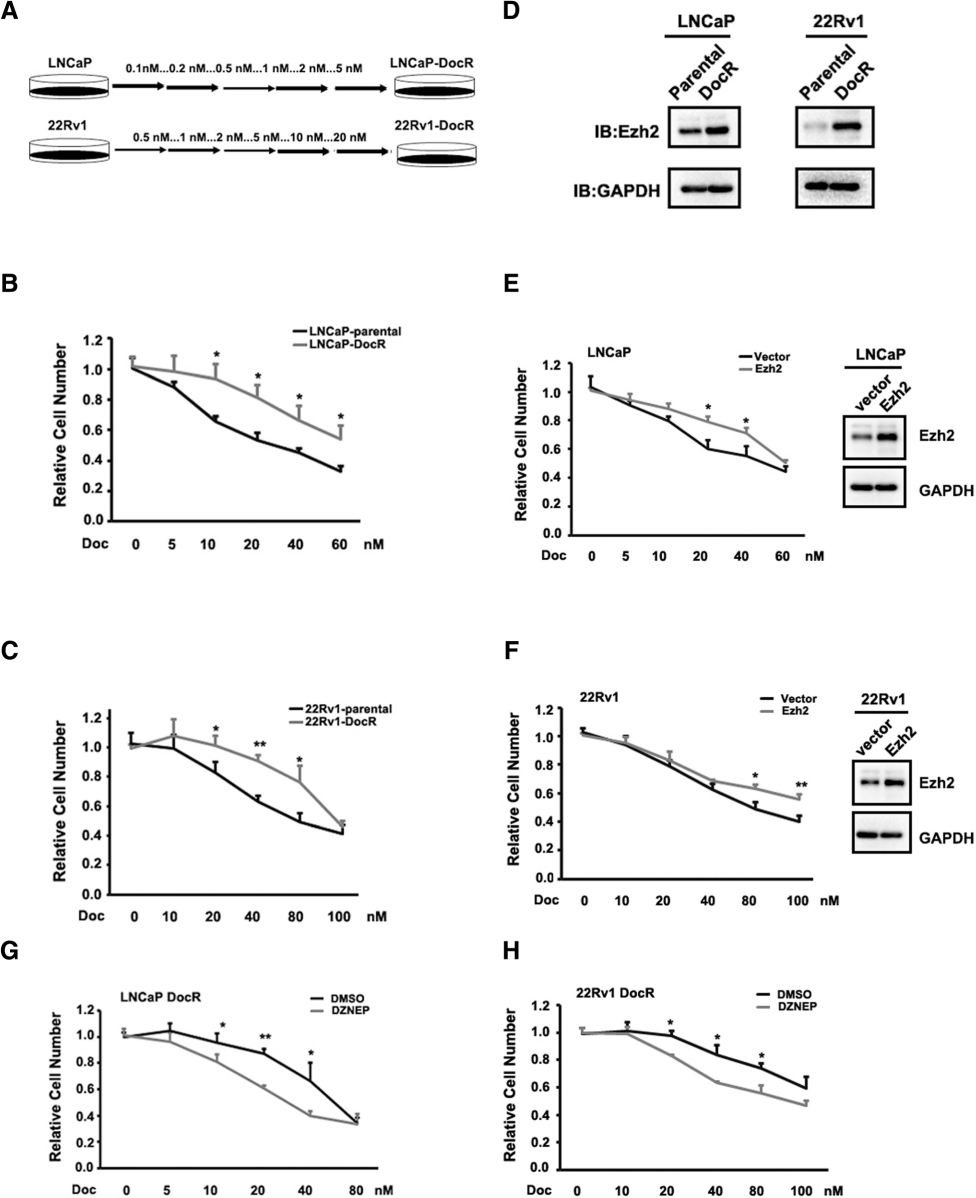
Ezh2 was required and sufficient to cause Doc resistance. **a** Cartoon showing how LNCaP DocR and 22Rv1 DocR cells were established. **b**, **c** Confirmation of Doc resistance phenotype of LNCaP cells (**b**) and 22Rv1 cells **c**. D. Ezh2 was induced in Doc resistant cells at both protein levels. E-F. Forced expression of Ezh2 was sufficient to cause Doc resistance in LNCaP cells (**e**) and 22Rv1 cells (**f**). Ezh2 inhibition by DNZEP could re-sensitize LNCaP DocR cells (**g**) and 22Rv1 DocR cells (**h**) to Doc treatment. 2 μM DNZEP was used and GAPDH was used as loading control. P* < 0.05; P** < 0.01

To explore the mechanisms that responsible for the development of Doc resistance, we established two Doc resistant cell lines (LNCaP DocR and CWR22Rv1 DocR) by continuously exposing them to Doc as indicated in Fig. 1a. The Doc resistance phenotype was confirmed by Fig. 1b, c, which indicated that cells from LNCaP DocR and CWR22Rv1 DocR had much more resistance as to various concentrations of Doc treatment compared with those from their parental cells.

Given the fact Ezh2 plays key role in determining androgen-dependent or androgen-independent growth of PCa [18], we tempted to test whether Ezh2 was altered in our Doc resistant cell lines. As shown in Fig. 1d, the protein levels of Ezh2 were dramatically elevated in both LNCaP DocR and CWR22Rv1 DocR cells compared to their corresponding parental cells. To test whether Ezh2 was a causal factor determining Doc resistance, we overexpressed Ezh2 in LNCaP and CWR22Rv1 cells and found that Ezh2-expressing cells had poor response to Doc treatment when compared to control cells (Fig. 1e, f). In addition, Ezh2 inhibition by small molecule, DZNEP or GSK126, had the capacity to re-sensitize LNCaP DocR cells (Fig. 1g and Additional file 1: Figure S1a) and CWR22Rv1 DocR cells (Fig. 1h and Additional file 1: Figure S1b) to Doc treatment. Collectively, these results indicate that Ezh2 was required and sufficient to cause Doc resistance.

### Cancer stem cells were highly enriched in DocR cells

**Fig. 2:**
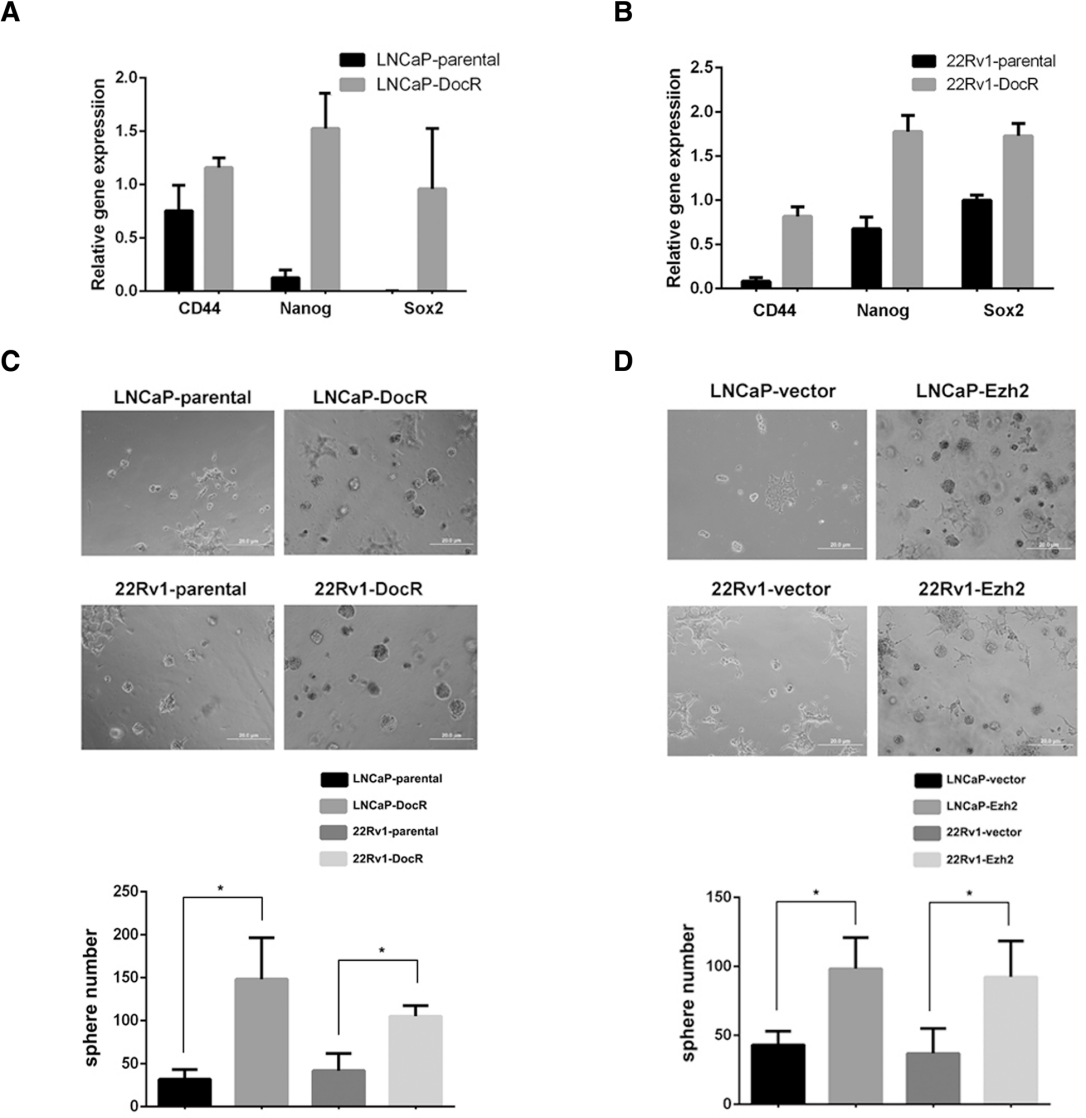
Cancer stem cells were highly enriched in DocR cells. A-B. qPCR results showed that cancer stem cell markers (CD44, Nanog, Sox2) were highly induced in LNCaP DocR cells (**a**) and 22Rv1 DocR cells (**b**) compared to their corresponding parental cells. GAPDH was used as control. **c**. Top, representative images showing that the population of cancer stem cells was enriched in LNCaP DocR and 22Rv1 DocR cells, monitored by sphere formation assay. Bottom, statistical analysis of spheres. **d**. Top, representative images revealing that Ezh2 overexpressing cells had more cancer stem cells compared to vector bearing cells. Bottom, statistical analysis of spheres. P* < 0.05

Interestingly, we found that cancer stem cell markers (CD44, Nanog, Sox2) were overexpressed in LNCaP DocR (Fig. 2a) and CWR22Rv1 DocR cells (Fig. 2b) compared to their parental cells. To confirm this finding, we performed sphere formation assay to check whether the population of cancer stem cells was indeed enriched in these two DocR cell lines. The result from sphere formation assay was consistent with the gene expression of cancer stem cell markers (Fig. 2c). Importantly, introduction of Ezh2 into LNCaP and CWR22Rv1 was sufficient to bestow cells with the properties of cancer stem cells (Fig. 2d and Additional file 2: Figure S2), which was consistent with previous publications [18, 19]. These data demonstrate that the induction of Ezh2 may be indispensable for the increased population of cancer stem cells.

### Ezh2 was indispensable for the increased population of cancer stem cells in doc resistant cells

**Fig. 3:**
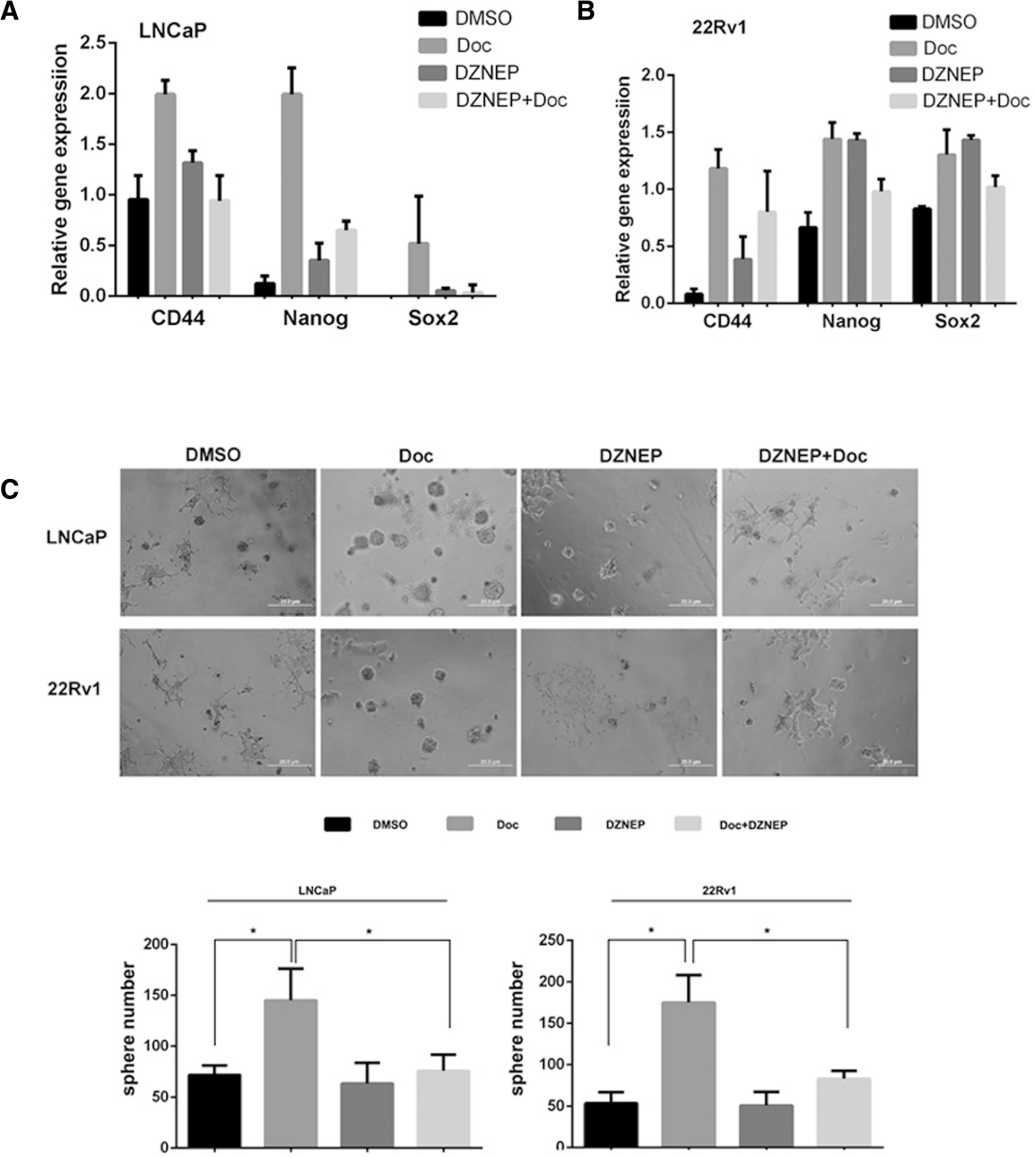
Ezh2 was indispensable for the increased population of cancer stem cells in Doc resistant cells. **a**, **b**. Inhibition of Ezh2 by DZNEP could reverse Doc-induced gene expression of cancer stem cell markers in LNCaP cells (**a**) and 22Rv1 cells (**b**). QPCR was performed after cells were treated with Doc (1 nM) or DNZEP (2 μM) for 2 days. **c**. Top, representative images showing that inhibition of Ezh2 by DZNEP could reverse Doc-induced enrichment of cancer stem cells in LNCaP and 22Rv1 cells. Bottom, statistical analysis of spheres. Sphere formation assay were conducted after cells were treated with Doc (1 nM) or DNZEP (2 μM) for 2 days

Given the fact that Ezh2 was an important player in determining the population of cancer stem cells and Ezh2 was overexpressed in our established Doc resistant cell lines, we hypothesized that Ezh2 was involved in the homeostatic regulation of cancer stem cells upon Doc treatment. First, we found that transient treatment of Doc for 2 days could increase levels of cancer stem cell markers including CD44, Nanog and Sox2 in both LNCaP cells and CWR22Rv1 cells (Fig. 3a, b). While these induction could be attenuated by DZNEP (a specific inhibitor of Ezh2) treatment (Fig. 3a, b). Importantly, the stronger sphere forming ability mediated by Doc treatment were still impaired by DZNEP treatment (Fig. 3c). The above evidence suggest that Ezh2 is required for Doc-induced cancer stem cells.

### MiR-101-3p and miR-138-5p were involved in doc resistance by targeting Ezh2

**Fig. 4:**
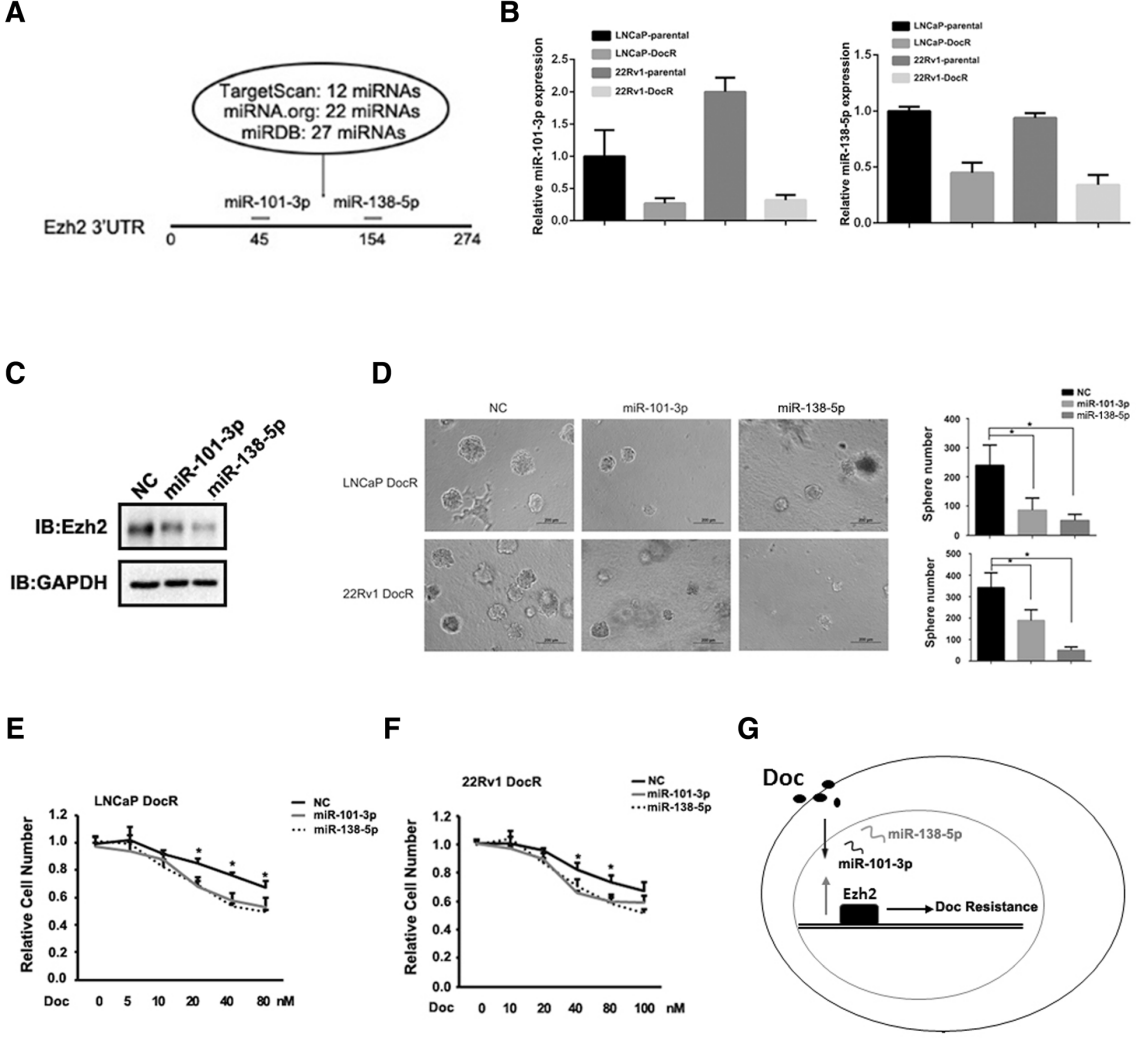
MiR-101-3p and miR-138-5p were involved in Doc resistance by targeting Ezh2 **a**. MiR-101-3p and miR-138-5p were predicated to target Ezh2. **b**. MiR-101-3p and miR-138-5p were reduced in LNCaP DocR cells and 22Rv1 DocR cells. **c**. MiR-101-3p and miR-138-5p could reduce Ezh2. GAPDH was served as loading control. **d**. MiR-101-3p and miR-138-5p inhibited the sphere forming ability of DocR cells. **e**, **f**. MiR-101-3p and miR-138-5p restored Doc sensitivity of LNCaP DocR cells (**e**) and 22Rv1 DocR cells (**f**). G. Mechanistic depiction of how Ezh2 plays role in the development of Doc resistance. P* < 0.05

Although Ezh2 protein levels were over-induced in our established DocR cells (Fig. 1d), its mRNA levels were indistinguishable between parental cells and DocR cells (data not shown), suggesting there is a post-transcriptional regulation on Ezh2. MiRNA-mRNA regulation represents one of mechanisms to regulate gene expression at post-transcriptional level so that we sought to explore whether miRNAs were involved in Ezh2 induction as well as the development of Doc resistance in DocR cells. We focused on miR-101-3p and miR-138-5p because they are predicated by three miRNA target programs to regulate the 3’UTR of Ezh2 (Fig. 4a). Of note, the expression levels of miR-101-3p and miR-138-5p were down-regulated in both LNCaP DocR and CWR22Rv1 DocR cells compared to their corresponding parental cells (Fig. 4b) and forced expression of these two miRNAs could reduce Ezh2 protein levels (Fig. 4c). Importantly, overexpression of miR-101-3p or miR-138-5p could restore Doc sensitivity (Fig. 4e, f) and impaired the sphere forming ability of DocR cells (Fig. 4d) in both LNCaP and CWR22Rv1 cells.

In summary, Ezh2 had an elevated levels in Doc resistant cells, which was mediated by the downregulation of miR-101-3p and miR-138-5p, determining the development of Doc resistance (Fig. 4g).

## Discussion

Doc resistance is a big obstacle in the treatment of metastatic CRPC. Previous investigations have demonstrated that several mechanisms accounted for Doc resistance. AR signaling, the central player in PCa progression, was involved in the development of Doc resistance. One suppressing action of Doc on PCa was to inhibit AR activity via blocking its nuclear translocation [20]. However, ARv7, one AR variant, was resistant to Doc treatment due to its constitutively nuclear accumulation [21]. In addition, the inductions of ABC transporter ABCB1, Bcl2, NFkB were also considered as mechanisms responsible for Doc resistance [22–24]. In this study, we found Ezh2 was required and sufficient to cause Doc resistance in PCa cells: overexpression of Ezh2 made PCa cells more resistant to Doc treatment while suppression of Ezh2 activity by its inhibitor DNZEP restored Doc sensitivity in Doc resistant PCa cells. Further study we found that Ezh2 could increase the population of cancer stem cells by regulating Nanog, Sox2, CD44. Inhibition of Ezh2 could reverse Doc-induced expression of Nanog, Sox2, CD44 and Doc-induced enriched population of cancer stem cells. In addition, reduction of miR-101-3p and miR-138-5p accounted for the overexpressed Ezh2 in DocR cells. Collectively, these data indicate that Ezh2 could become a therapeutic target for Doc resistant PCa.

Doc is a chemotherapy drug to treat PCa patients. The mechanism of Doc action is to disrupt cell invasion by stabilizing microtubule structure [9]. Although we observed a dramatic induction of Ezh2 in DocR cells at protein levels, its mRNA levels were indistinguishable between Doc sensitive cells and Doc resistant cells, suggesting there is post-transcriptional or post-translational regulation on Ezh2 upon Doc treatment. Indeed, two miRNAs (miR-101-3p and miR-138-5p) were downregulated in DocR cells, which targeted Ezh2 mRNA for degradation. Some miRNAs are characterized by their blockage of mRNA translation without altering transcript levels [25]. The expression levels of miR-101-3p and miR-138-5p were silenced, probably due to the epigenetic regulation on their promoters in our Doc resistant cells, so that Ezh2 protein levels were dramatically enhanced. In addition, our data was consistent with previous finding demonstrating that miR-101-3p could target Ezh2 via base-pairing with its 3’-UTR [26], suggesting targeting Ezh2 via introducing miRNAs may provide a therapeutic strategy to cure PCa patients.

As an epigenetically regulatory factor, Ezh2 was considered to silence gene expression by tri-methylating histone 3 lysine 27 (H2K27me3) via interacting with SUZ12, EED and RbAp46/48 [13]. Interestingly, Ezh2 could also up-regulate gene expression by di-metylating histone lysine 36 (H3K36me2) [27, 28]. Here, we showed that Ezh2 inhibition by DZNEP could reduce Doc-induced gene expression of cancer stem cell markers (Nanog, CD44 and Sox2), supporting the notion that Ezh2 acts on these genes independent of PRC2 complex. It has been reported that Ezh2 interacts with AR to regulate gene expression, providing survival signals to CRPC cells [15]. As an activator, Ezh2 requires its intact methyltransferase activity and the S21 phosphorylation mediated by Akt [15]. Thus, we postulate that Akt activity is also elevated in our Doc resistant cells, which is indispensable for the enzymatic activation of Ezh2.

## Conclusions

Ezh2 was overexpressed in our Doc resistant PCa cells and targeting Ezh2 with its inhibitor could overcome Doc resistance. Future directions would be focused on the mechanisms of how Ezh2 was induced. In addition, combinational therapy using Ezh2 inhibitor and anti-androgen would better suppress PCa progression.

## Additional files

Additional file 1: **Figure S1.** Ezh2 inhibitor GSK126 restored Doc sensitivity in LNCaP DocR (A) and 22Rv1 DocR (B) cells were established. 5 μM GSK126 was used. P* < 0.05; P** < 0.01. (JPG 105 kb)

Additional file 2: **Figure S2.** The expression levels of stem cell markers were altered by Ezh2 overexpression in LNCaP (A) and 22Rv1 (B) cells. Gene expression was normalized to GAPDH. (JPG 111 kb)

## Abbreviations

ADT: Androgen deprivation therapy
CRPC: Castration resistant prostate cancer
Doc: Docetaxel
DocR: Docetaxel resistant
PCa: Prostate cancer
