# Supplementary figures and images for: Targeting Ezh2 could overcome docetaxel resistance in prostate cancer cells
Source: BMC Cancer. 2019 Jan 8;19:27. doi: 10.1186/s12885-018-5228-2 (PMC6324167; doi:10.1186/s12885-018-5228-2)

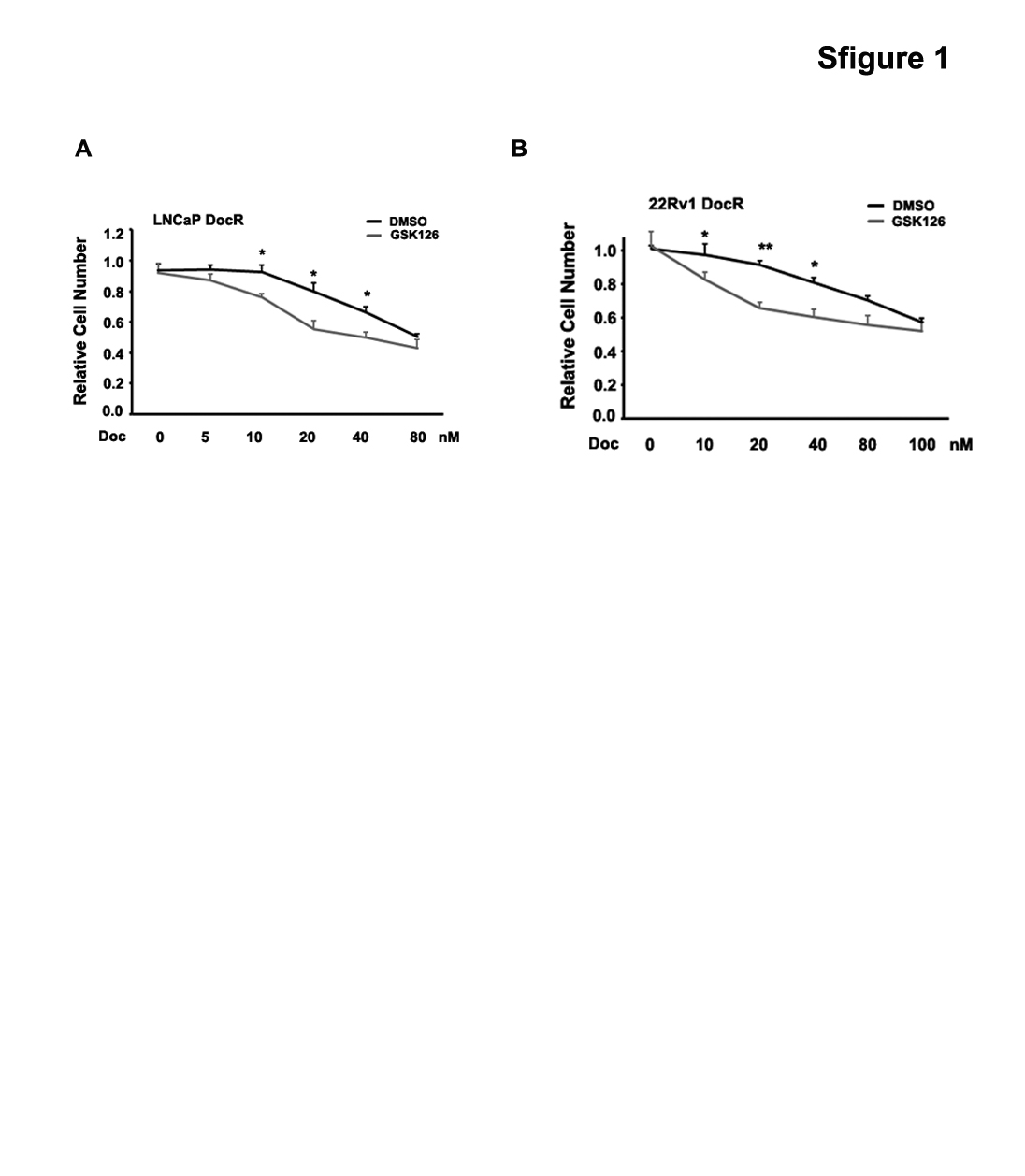

Supplement: Supplementary file 1 — Figure S1. Ezh2 inhibitor GSK126 restored Doc sensitivity in LNCaP DocR (A) and 22Rv1 DocR (B) cells were established. 5 μM GSK126 was used. P* < 0.05; P** < 0.01. (JPG 105 kb) [file 12885_2018_5228_MOESM1_ESM.jpg]

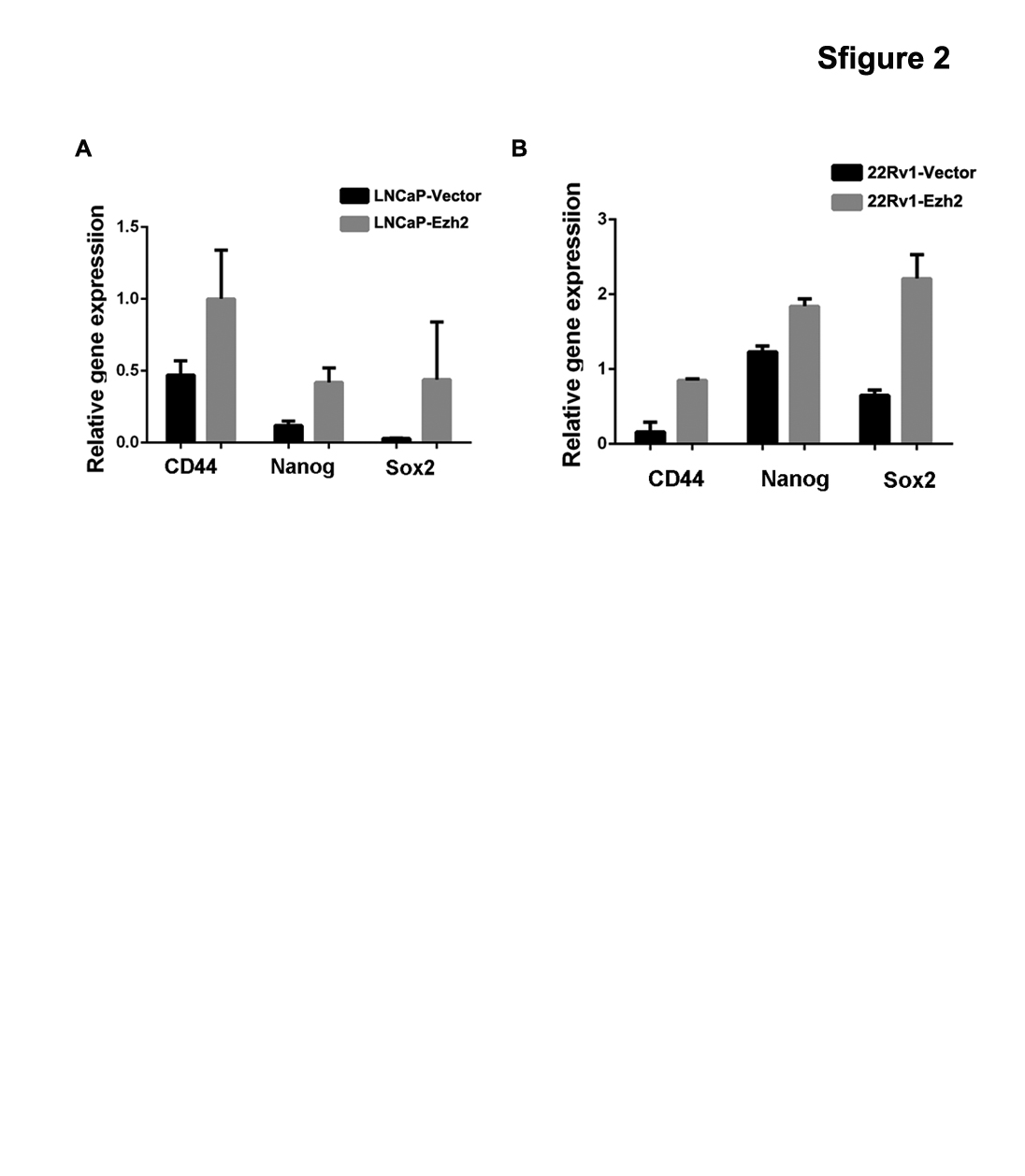

Supplement: Supplementary file 2 — Figure S2. The expression levels of stem cell markers were altered by Ezh2 overexpression in LNCaP (A) and 22Rv1 (B) cells. Gene expression was normalized to GAPDH. (JPG 111 kb) [file 12885_2018_5228_MOESM2_ESM.jpg]
